# Intraventricular Pleomorphic Xanthoastrocytoma: A Case Report and Systemic Review

**DOI:** 10.7759/cureus.52510

**Published:** 2024-01-18

**Authors:** Xiaotong Wu, Kota Yokoyama, Kazutaka Sumita, Yoji Tanaka, Ukihide Tateishi

**Affiliations:** 1 Radiology, Tokyo Medical and Dental University, Tokyo, JPN; 2 Endovascular Surgery, Tokyo Medical and Dental University, Tokyo, JPN; 3 Neurosurgery, Tokyo Medical and Dental University, Tokyo, JPN

**Keywords:** intraventricular pxa, corpus callosum (cc) agenesia, anaplastic pleomorphic xanthoastrocytoma, brain tumor, mri, pleomorphic xanthoastrocytoma

## Abstract

We present a unique case of a 45-year-old male with cerebral palsy, who experienced walking difficulties and altered consciousness. The initial MRI revealed an intraventricular mass that rapidly enlarged over a month, consisting of two distinct components with different characteristics on CT and MRI, and was associated with agenesis of the corpus callosum. Despite initial treatment, surgical intervention was necessary, where preoperative imaging suggested an exophytically growing glioblastoma. However, postsurgical pathological examination identified the mass as pleomorphic xanthoastrocytoma (PXA), World Health Organization (WHO) Classification of Tumours of the Central Nervous System (CNS) grade 3. This study is notable for its rarity and complexity, challenging standard diagnostic approaches.

PXA is an uncommon astrocytic tumor, and its occurrence intraventricularly is extremely rare. This study highlights its unique imaging features and the critical role of MRI in preoperative assessment, underlining the tumor's unusual intraventricular location, and its relationship with corpus callosum agenesis. Our comprehensive review of PXA's history and imaging spectrum offers valuable insights for neuroradiologists and neurosurgeons, emphasizing the diagnostic challenges of such rare tumor locations and the importance of meticulous MRI analysis for accurate diagnosis.

## Introduction

Pleomorphic xanthoastrocytoma (PXA) is classified as circumscribed astrocytic gliomas in the 2021 World Health Organization (WHO) Classification of Tumours of the Central Nervous System (CNS) fifth edition [1]. It represents a distinct subset of astrocytoma, accounting for less than 0.3% of primary CNS tumors [2].

While PXA is uncommon, its occurrence within the ventricular system is exceedingly rare [3]. This unique location presents diagnostic and management challenges, offering an opportunity to explore nuanced imaging characteristics specific to ventricular PXAs.

In this study, we present a case of intraventricular PXA, CNS WHO grade 3, focusing on its imaging characteristics, particularly on magnetic resonance imaging (MRI). Preoperative diagnosis of intraventricular PXA is challenging but crucial for guiding surgical decisions, determining treatment approaches, and directing genetic testing, all of which contribute to more precise and effective patient management. By reviewing previous reports, we aimed to provide a comprehensive understanding and insights that could aid neuroradiologists in diagnosis.

## Case presentation

**Figure 1:**
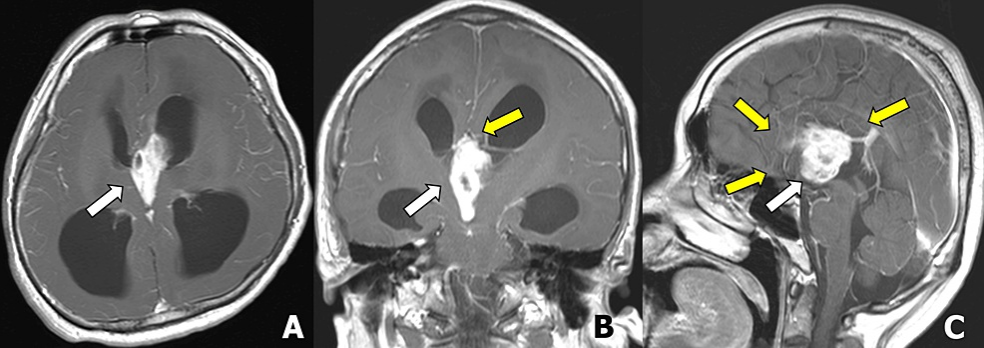
Initial brain ｍagnetic resonance imaging. Contrast-enhanced T1-weighted images show an intraventricular mass in the third ventricle to the anterior horn of the left lateral ventricle (A-C; white arrow) with hydrocephalus. Corpus callosum agenesis is noted in the coronal and sagittal images (B and C; yellow arrows).

A 45-year-old male patient with cerebral palsy, who was previously independent in basic activities, presented with difficulty walking for the past seven days. He developed altered consciousness, prompting his family to bring him to the emergency department. Initial treatment for suspected viral meningitis did not lead to improvement, and an initial MRI revealed an intraventricular mass measuring 52×22 mm and characterized as a heterogeneous enhancing tumor with cystic components, along with agenesis of the corpus callosum (Figures 1A-1C). The patient had increased intracranial pressure due to the intracranial mass and he was treated with dexamethasone. As a result, his altered consciousness improved, but his motor function remained impaired. He was subsequently transferred to our hospital for surgical treatment.

**Figure 2:**
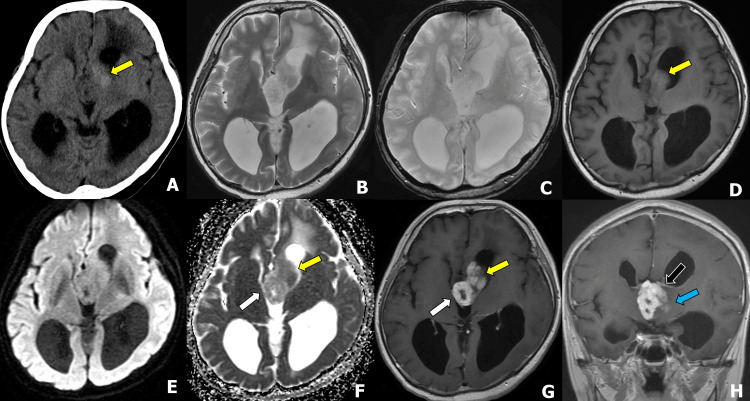
Preoperative CT and MRI one month after the initial visit. (A) Axial computed tomography (CT), (B) T2-weighted image (T2WI), (C) T2*WI, (D) T1WI, (E）diffusion-weighted image (DWI), (F) apparent diffusion coefficient (ADC) map, (G) axial postcontrast T1WI, and (H) coronal postcontrast T1WI. The tumor consisted of two following components: the ventral component appeared dense on CT, slightly hyperintense on T1WI, demonstrated contrast enhancement on postcontrast T1WI, and had a decreased ADC value (F; yellow arrow). The dorsal component showed ring enhancement on postcontrast T1WI and an increased ADC value in the solid component (E and F; white arrows). In DWI, both components showed signals close to the cerebral white matter, and no marked high signals were observed. No hemorrhage or calcification was observed on T2*WI (C). The tumor predominantly occupies the third ventricle and left Moro foramen, located beneath the thalamostriate vein (H; black arrow), and appears contiguous with the brain parenchyma (H; blue arrow).

Preoperative computed tomography (CT) and MRI performed one month later showed an enlarged tumor (Figures 2A-2H). The tumor consisted of two following components: the ventral component appeared dense on CT, slightly hyperintense on T1-weighted images (T1WI), demonstrated contrast enhancement on postcontrast T1WI, and had a decreased apparent diffusion coefficient (ADC) value (Figure 2, yellow arrows). In the diffusion-weighted image (DWI), both components showed signals similar to cerebral white matter, with no marked high signals. The dorsal component showed ring enhancement on postcontrast T1WI, and an increased ADC value in the solid component (Figure 2, white arrows). No hemorrhage or calcification was observed on T2*WI (Figure 2C). The tumor predominantly occupies the third ventricle and left Moro foramen, located beneath the thalamostriate vein (Figure 2H, black arrow), and appears contiguous with the brain parenchyma (Figure 2H, blue arrow). Therefore, an exophytically growing high-grade glioma, such as glioblastoma, was considered. Ependymomas were also included in the differential diagnosis, but the rapid growth observed was atypical for circumscribed low-grade gliomas, such as pilocytic astrocytoma or angiocentric gliomas.

**Figure 3:**
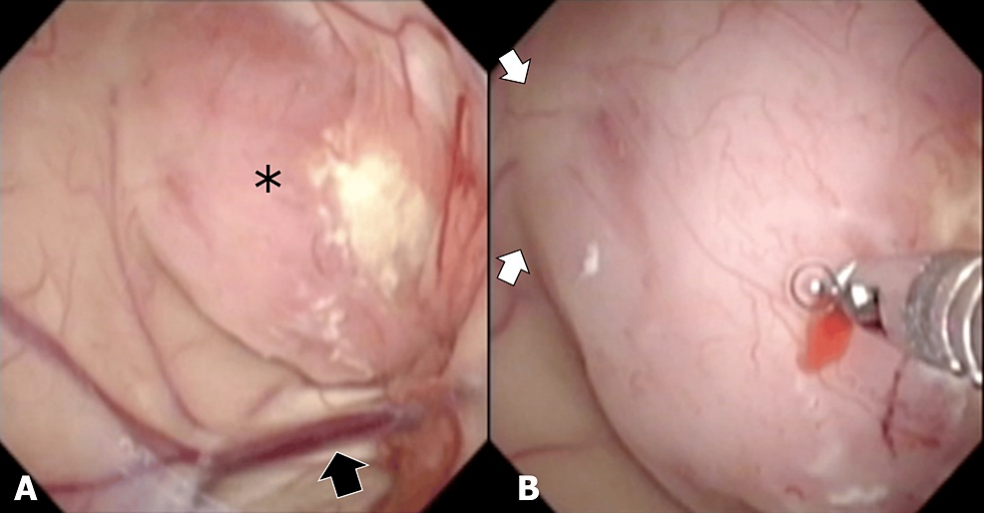
Intraoperative endoscopic images. Intraoperative endoscopic images viewed from the frontal horn of the left ventricle showed a tumor (*) located beneath the brain parenchyma and near the left thalamostriate vein (A; white arrow). A gentle elevation, indicative of an intraaxial tumor, arises from the left frontal lobe (B; white arrows). The tumor nearly occludes the foramen of Monro. Resection of the tumor surface was performed using tumor forceps, with careful avoidance of the tumor's red area.

An endoscopic biopsy was performed, and intraoperatively a tumor bulging into the ventricles was observed, with a gentle elevation from the brain parenchyma, indicating it as an intraaxial tumor, which led to the diagnosis of low-grade glioma (Figures 3A, 3B).

**Figure 4:**
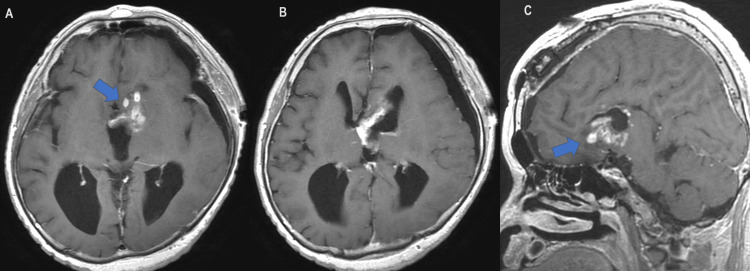
Postoperative MRI of the patient. Contrast-enhanced T1-weighted images display a reduced tumor in the ventricles (A and B) and improved hydrocephalus, with the mass's decreased size more clearly indicating its left frontal basal origin (A and C; arrows).

Given the preoperative imaging suspicion of high-grade glioma, tumor resection was undertaken. The surgeon approached from the component in the left foramen of Monro and proceeded to resect the soft, hemorrhagic mass. Inside the cystic component, the walls of the third ventricle were visible and clear of adhesions or infiltration. Postoperative MRI showed a reduced intraventricular mass volume, clarifying that the mass protruded from the base of the left frontal lobe into the ventricles (Figures 4A-4C; arrows).

**Figure 5:**
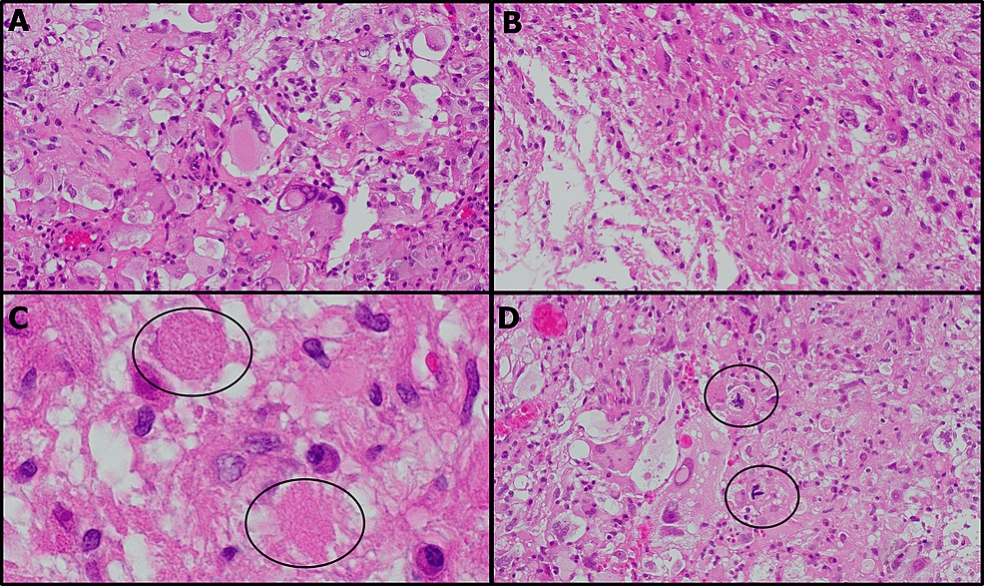
H&E stained histology images of the excised specimen. The histological specimen displayed characteristics of pleomorphic xanthoastrocytoma. Most nuclei were enlarged and exhibited bizarre shapes, accompanied by a vacuous, foamy cytoplasm (A). A significant number of xanthic cells were observed (B); numerous eosinophilic granular bodies were also visible (C). Mitotic figures are present (D).

The histological specimen revealed characteristics consistent with PXA (Figures 5A-5D). Upon hematoxylin and eosin (HE) staining, it showed large and irregular nuclei demonstrating pleomorphism. The specimen also included xanthic cells with a notably vacuolar or foamy cytoplasm. Additionally, degenerative structures, specifically eosinophilic granular bodies, and 6 mitotic figures over 10 high power fields were observed. The final diagnosis was pleomorphic astrocytoma, CNS WHO grade 3. The patient was transferred to a convalescent hospital 1.5 years later while receiving postoperative radiation therapy (60 Gray in 30 fractions) and temozolomide, with no sign of lesion growth during this period.

## Discussion

PXA was first reported by Kepes et al. in 1979 and was introduced to the 1993 WHO tumor classification of CNS as an individual diagnosis, and was classified as WHO grade 2 [4]. However, 15-50% of PXAs featured necrosis, hypervascularity, and mitotic activity, and thus in the revised third edition (2007), the group of PXAs with more invasive clinical behavior and histological features was classified as a novel subtype of PXA with anaplastic features [5]. In 2016, the fourth edition additionally introduced anaplastic PXA [6], whereas in the fifth edition (2021), the anaplastic PXA was renamed as PXA, CNS WHO grade 3 [1]. This reflects an understanding of the more aggressive nature of these tumors.

PXA, a rare tumor accounting for less than 0.3% of primary CNS tumors, affects individuals regardless of gender and typically presents in children and young adults, with a mean diagnosis age of 26.3 years [7]. Cases in the elderly have also been observed [8].

The pathogenesis of PXAs remains unknown. However, an association between PXA and type 1 neurofibromatosis has been reported, which is related to the high-frequency mutations in mitogen-activated protein kinase (MAPK) signaling pathway genes in PXA [3]. A small number of cases have also reported the associations between PXA a familial melanoma-astrocytoma syndrome (with CDKN2A inactivation) [9].

On imaging, PXA may show various appearances on CT (low density, high density, or mixed density), and shows heterogeneous enhancements. On the other hand, in MRI, the solid portion of PXAs normally shows iso-to-low signal intensity on T1WI, high or heterogeneous signal intensities on T2WI, and moderate to strong gadolinium enhancement, often with inconspicuous edemas [10]. Collectively, these were consistent with the imaging features of the present case. The exact cause of the cystic formation remains uncertain. During the surgery, we approached the tumor via the left foramen of Monro, which restricted our ability to perform a detailed macroscopic comparison. We found fluid in the cyst but no extensive necrosis or hematoma. The cystic component of the tumor had notably thin walls, allowing the underlying ventricular walls to be seen through them endoscopically. This suggests that the cyst formation was due to fluid accumulation inside the tumor, likely caused by internal necrosis in the parts of the tumor furthest from its origin due to inadequate blood supply. Adjacent to the left frontal lobe, the tumor showed higher CT density and lower ADC values, indicative of variable cellularity, likely affected by the abundance of nutritive blood vessels. This led to a unique morphology consisting of two distinct masses, an exceedingly rare presentation for PXA.

Most PXAs (98%) originate in the supratentorial region, particularly the temporal lobe [3]. Infratentorial PXAs are rare, but cases in the cerebellum, spinal cord, and even the retina in children have been reported. Based on the up-to-date publications, there are 11 cases with confirmed image data for intraventricular PXAs, of which five were classified as grade 2 and six were classified as grade 3 (Table 1) [10-19].

**Table 1:** Up-to-date publications with confirmed image information of intraventricular PXAs. PXA: pleomorphic xanthoastrocytoma; DWI: diffusion-weighted image; T2WI: T2-weighted image

S. no.	Previous reports	Grade	Age	Gender	Location	Imaging pattern	T2WI	DWI	Contrast enhancement	Corpus callosum agenesis
1	Menendez et al. (2014) [11]	2	24	Male	Third ventricle	Solid	N/A	N/A	Homogeneous	No
2	Abe et al. (2006) [12]	2	41	Female	Hypothalamus and third ventricle	Solid	N/A	N/A	Homogeneous	No
3	Yang et al. (2012) [13]	2	42	Female	Right lateral ventricle	Solid-cystic	N/A	N/A	Heterogenous	No
4	Liu et al. (2019) [14]	2	28	Male	Left lateral ventricle	Solid-cystic	Heterogeneous	N/A	N/A	No
5	Yan et al. (2018) [10]	2	25	Female	Right lateral ventricle	Solid	N/A	Hyperintense	Homogeneous	Yes
6	Bettencourt et al. (2023) [15]	3	33	Male	Lateral and third ventricles	Cystic-necrotic	Hyperintense (solid component)	Hyperintense (solid component)	Heterogenous	No
7	She et al. (2018) [16]	3	41	Female	Right lateral ventricle	Solid-cystic	Heterogeneous	N/A	Heterogenous	No
8	Rodriguez-Mena et al. (2012) [17]	3	54	Male	Right parieto-occipital lobe and ipsilateral ventricle	Multi-cystic	N/A	N/A	Vivid enhancement	No
9	Fu et al.(2010) [18]	3	52	Male	Right lateral ventricle and third ventricle	Solid-cystic	Hyperintense (solid component)	N/A	Heterogenous	No
10	Roberti and Baggenstos (2018) [19]	3	65	Female	Septum pellucidum and lateral ventricles	Solid-cystic	N/A	N/A	Heterogenous	Yes
11	Yan et al. 2018 [10]	3	23	Female	Right lateral ventricle	Solid-cystic	N/A	Hyperintense	Heterogenous	No

In the present case, the tumor was located not only in the cerebral ventricles but also consisted of two masses, which is exceedingly rare for PXA. PXAs predominantly present as solid tumors, with approximately two-thirds being predominantly solid and one-third predominantly cystic with or without mural nodules, often accompanied by peritumoral edema [10]. However, in the present case, the tumor exhibited two different masses with solid and cystic components. Moreover, the tumor occupied the third ventricle and the foramen of Monro, leading to hydrocephalus. Concurrently, agenesis of the corpus callosum, potentially associated with cerebral palsy, was noted [20]. This anomaly may have influenced the tumor's growth direction towards the ventricles of the brain, rather than its usual growth on the brain's surface. This is because, usually, the genu of the corpus callosum is interposed between the presumed site of origin at the base of the frontal lobe and the ventricles (Figure 4). Therefore, the absence of the corpus callosum in this case likely had a significant impact on the intraventricular growth of the tumor. Notably, in past reports and including this case, agenesis of the corpus callosum was found in three out of 12 intraventricular PXA cases. In essence, a structural defect in the corpus callosum could be a significant factor in the intraventricular occurrence of PXAs, especially considering its higher incidence in comparison with the general population. While a definitive correlation between agenesis of the corpus callosum and the incidence of brain tumors has not been established, it is recognized that this condition is often associated with various malformations and genetic abnormalities [21]. Some of those syndromes have been reported in association with increased incidence of benign and malignant tumors [22]. If abnormalities occurring during the same developmental period are implicated, there may be a relationship between the two conditions. In the present case, while no specific genetic diseases or malignancies other than the brain tumor have been identified, the emergence of a rare tumor suggests the possibility of underlying genetic abnormalities, which cannot be entirely ruled out.

Pathologically, PXA usually presents as large pleomorphic, multinucleated cells, including spindle cells and lipidized cells, accompanied by depositions of numerous eosinophilic granular bodies and reticulin. Genetically, the PXA is characterized by BRAF p.V600E mutations (or mutations in other MAPK signaling pathway genes), and homological loss in CDKN2A and/or CDKN2B [23]. The grading is based on necrosis and a number of mitotic features. There were some mitotic nuclei in the present case and thus it was diagnosed as PXA CNS WHO grade 3. Since the tumor was excavated for removal, a direct comparison between the imaging findings and the macroscopic appearance was not possible. Although the reasons for the cystic formation depicted in the MRI could not be definitively determined, as mentioned earlier, internal necrosis is likely related. It's important to note that all reported cases of grade 3 intraventricular PXAs have shown cystic components, and it is reasonable to assume that the rapid growth associated with this CNS WHO grade 3 tumor may have played a significant role.

While PXA, WHO grade 2, generally shows a favorable prognosis with a 10-year survival rate of over 70%, progression and recurrence are notable risks, emphasizing the importance of early and complete surgical resection. In contrast, PXA, WHO grade 3, seen in 10-20% of cases, represents a more severe progression, often necessitating additional treatments such as radiotherapy and chemotherapy. This form of PXA, which can develop from a progression of WHO grade 2 or de novo, is associated with a less favorable prognosis, as evidenced by a five-year survival rate of approximately 57.1% [10]. This underlines the critical need for vigilant management and follow-up in these cases.

## Conclusions

In this study, we discussed a rare instance of intraventricular PXA, CNS WHO grade 3, with distinct imaging characteristics and co-existing agenesis of the corpus callosum. The tumor's unusual presentation, including its location and dual mass structure, emphasizes the importance of detailed MRI analysis in diagnosis. This study contributes valuable insights into the diagnosis, pathology, and management of this rare CNS tumor, enhancing the knowledge base for neuroradiologists and neurosurgeons.
